# Editorial: Innovative non-thermal technologies for the extraction and modification of bioactive compounds from food processing by-products

**DOI:** 10.3389/fnut.2023.1161957

**Published:** 2023-03-20

**Authors:** Zhi-Hong Zhang, Lang-Hong Wang, Jing-Kun Yan, Rana Muhammad Aadil

**Affiliations:** ^1^School of Food and Biological Engineering, Jiangsu University, Zhenjiang, China; ^2^Guangdong Provincial Key Laboratory of Intelligent Food Manufacturing, Foshan University, Foshan, China; ^3^Engineering Research Center of Health Food Design & Nutrition Regulation, Dongguan Key Laboratory of Typical Food Precision Design, China National Light Industry Key Laboratory of Healthy Food Development and Nutrition Regulation, School of Life and Health Technology, Dongguan University of Technology, Dongguan, China; ^4^National Institute of Food Science and Technology, University of Agriculture, Faisalabad, Pakistan

**Keywords:** non-thermal technologies, extraction, modification, bioactive compounds, food processing

At present, many food processing by-products are produced in the food industry and most of them are directly discarded, which results in the waste of resources and causes potential environmental pollution. According to the reports, about 50% of fruits and vegetables becomes by-products during its processing (1, 2). However, food processing by-products are rich in bioactive substances (polyphenols, polysaccharides, proteins). As compared to traditional thermal technologies, non-thermal technologies have the advantages of less solvent utilization, mild conditions and a high extraction rate, which has become an effective method for the extraction of bioactive compounds (3–5). For example, the demand for vegetables and fruits with fresh-like qualities encourages the use of high-pressure processing (HPP) in commercial industries, since it can preserve the food's original organoleptic qualities while also having a preservation impact [Xia et al.; (6, 7)]. Similarly, ultrasound has been extensively researched in the food industry by extracting substances of interest, such as natural pigments which are in high demand across a variety of industries, not only for their aesthetic qualities but also for their health benefits, compliance with regulations, and evolving consumer tastes (Linares and Rojas). Ultrasonic-assisted dual-alkali pretreatment and enzymatic hydrolysis is other innovative technique, which can help in better fermentation yields (Gai et al.). Other relevant methods for the food sector include wet heating millard reactions and pulsed electric field (PEF) treatment (Yang et al.; Yan et al.). In addition, these techniques utilized moderate energy. So, these techniques can change the weak non-polar covalent bonds in the biological macromolecules, such as hydrogen bonds, hydrophobic bond force, van der Waals force, etc., thereby could change the spatial structure of bio-macromolecules, such as polysaccharides and proteins, and then change their physicochemical properties and processing characteristics, so it has become an innovative physical modification method to improve the biological properties or processing characteristics of recovered bioactive compounds (5, 8, 9).

In a mini-review by Xia et al., the authors summarize the major challenges of HHP and introduced some of the recent advances in HHP-based combination strategies for microbial safety (combination of high inactivation efficiencies). Considerations and requirements about the future process design and development of HHP-based combination technologies were discussed.

In a review article, the authors presented the effect of ultrasound-assisted extraction (UAE) on the extraction of pigments, and critically analyze the application of UAE to obtain natural pigments (carotenoids, chlorophyll, anthocyanins, and betalains) from different food by-products. Moreover, relevant information, which may be useful to compare the specific energy consumption between UAE and other extraction technologies; this can lead to an expansion of the UAE treatments toward exploration and optimization of parameters for natural pigments extraction (Linares and Rojas).

In a research article, the authors mainly focused on ultrasonic-assisted dual-alkali pretreatment and enzymatic hydrolysis of sugarcane bagasse followed by *Candida tropicalis* fermentation to produce xylitol. The results showed that the highest xylose content of 1.928 g/L was obtained under the condition of ultrasonic-assisted NaOH, and ammonia water mixed with alkali pretreatment. Moreover, the content of xylose reaches 2.431 g/L under the optimal parameter by Box–Behnken experimental design (BBD) of the response surface methodology (RSM), which may be due to the external appearance of the structure of sugarcane bagasse became more folds and furrows by ultrasonic-assisted alkaline pretreatment. In addition, an optimal increase in the ratio of enzymatic hydrolysate of sugarcane bagasse (3 times) could increase the xylitol yield by 55.42% (Gai et al.).

In a research article, the authors investigated the effect of pulsed electric field (PEF) pretreatment in combination with phosphorylation on the following parameters i.e., structure and immunoglobulin (Ig) G/IgE-binding ability of ovalbumin (OVA). They stated that the combined treatment of phosphorylation and PEF has reduced the IgG- and IgE-binding abilities. Moreover, they also suggested that PEF pretreatment had improved the phosphorylation of OVA as well as enhanced the reduction of IgG/IgE-binding capacity of phosphorylated OVA. In conclusion, combined treatment (PEF with phosphorylation method) was applied to modify the structure of OVA, thereby reducing the potential allergic reaction caused by hen egg white and improving the food safety of hen eggs (Yang et al.).

In a research article, authors have determined the effect of mixed-process methods on the ruminal degradability of whole cottonseed (WCS) both *in situ* and *in vitro*, and the effect on the production performance of dairy cows. The results indicated that the CA2 treatment group (crush-alkali 2: 2% NaOH+2% CaO) showed the highest ruminal degradation of WCS *in situ* and the highest intestinal digestibility of WCS *in vitro* compared to other treatment groups. Moreover, the dry matter intake, 4% fat-corrected milk production, milk protein, milk fat, and content of short-chain saturated fatty acid of milk in the CA2 group were significantly higher (*P* <0.05) than without the WCS group in an animal experiment of Holstein dairy cow for 60 d. Furthermore, dry matter intake (DMI), the conjugated linoleic acid (CLA) was significantly greater (*P* < 0.05) in the CA2 group than the other groups. Additionally, the free gossypol concentration in serum or milk was under safety level in the three groups. Overall, crush and alkalization (NaOH: CaO = 1:1) treatment could improve the utilization of WCS in dairy farms. This study provides a feasible solution for the application of WCS in dairy cow feed, which not only reduces the waste of resources but also improves the utilization rate of WCS (Sun et al.).

Yan et al. have investigated the effects of the molecular structure and emulsifying properties of ovalbumin (OVA) by wet heating Maillard reaction with three types of monosaccharides (i.e., xylose, glucose, and galactose). Results showed that increasing reaction temperature from 55 to 95°C could significantly improve the degree of grafting (DG), while glycosylated OVA conjugate with xylose at 95°C processed the highest DG of 28.46%. This reaction was further confirmed by the browning intensity determination. Analysis by Fourier transform infrared spectrophotometer, circular dichroism, and fluorescence spectra indicated that there were slight changes in the subunits and the conversion of α-helices to β-sheets, as well as the unfolded structures, thereby increasing the surface hydrophobicity and absolute zeta potential of obtained glycosylated OVA. Glycosylation endowed OVA with better emulsifying properties, especially the xylose glycosylated OVA was superior to that of glucose and galactose glycosylated OVA, which was mainly due to its shorter molecular chains with smaller steric hindrance for reaction. Furthermore, the enhancement of emulsifying properties may be attributed to the synergistic effect of stronger electrostatic repulsion of larger absolute zeta potential and the steric hindrance from thicker adsorbed layer, thereby inhibiting aggregation and flocculation of emulsion droplets.

Wang et al. stated that soluble dietary fiber (SDF) and selenium have been proven to be effective in preventing and relieving inflammatory bowel disease (IBD). They investigated and compared the therapeutic efficacy of millet-derived silylated-soluble dietary fiber (SeSDF) against dextran sulfate sodium (DSS)-induced colitis in mice alone or through the synergistic interaction between selenium and SDF. In female mice, Se-SDF markedly alleviated body weight loss, decreased colon length, reduced histological damage scores, and enhanced IL-10 expression to maintain the barrier function of intestinal mucosa compared to male mice. The 16S rRNA sequence analysis further indicated that pretreatment with SeSDF restored the gut microbiota composition in female mice by increasing the relative abundance of Lactobacillus and the Firmicutes/Bacteroidetes ratio. In conclusion, these findings demonstrated that Se-SDF can protect against DSS-induced colitis in female mice by regulating inflammation and maintaining gut microbiota balance. This study, therefore, provides new insights into the development of Se-SDF as a supplement for the prevention and treatment of colitis.

We think papers published on this Research Topic make an important contribution toward the advancement of knowledge and application of non-thermal techniques in different food products. We hope that this Special Issue can not only contribute to science but more importantly to the application of non-thermal techniques at an industrial scale.

## Author contributions

Z-HZ: investigation and writing—review and editing. L-HW and J-KY: writing—review and editing. RA: writing—original draft and writing—review and editing. All authors contributed to the article and approved the submitted version.
